# Fourth Ventricle Tumors: A Review of Series Treated With Microsurgical Technique

**DOI:** 10.3389/fsurg.2022.915253

**Published:** 2022-06-06

**Authors:** Rinat Sufianov, David Pitskhelauri, Andrey Bykanov

**Affiliations:** ^1^ Department of Neurooncology, N. N. Burdenko National Medical Research Center of Neurosurgery, Ministry of Health of the Russian Federation, Moscow, Russia; ^2^ Department of Neurosurgery, Sechenov First Moscow State Medical University (Sechenov University), Moscow, Russia

**Keywords:** IV ventricle, tumors of the IV ventricle, median suboccipital approach, minimally invasive suboccipital approach, posterior fossa, keyhole approach, brainstem

## Abstract

Tumors of the IV ventricle represent 1–5% of all intracranial lesions; they are implicated in 2/3 of the tumors of the ventricular system. According to modern standards, the first treatment stage for this pathology is microsurgical removal. Currently, for the removal of neoplasms of the IV ventricle and brainstem, the median suboccipital approach is widely used, followed by one of the microapproaches. Moreover, with the development of microsurgical techniques, keyhole approaches are now beginning to be utilized. However, surgical treatment of these tumors remains a challenge for neurosurgeons due to the proximity of functionally important anatomical structures (the brainstem, the cerebellum, pathways, vessels, etc.) of the posterior cranial fossa. Therefore, surgery in this area is associated with the possible occurrence of a wide range of postoperative complications. The authors provide a review of series of fourth ventricle tumors treated with microsurgical technique.

## Introduction

Tumors of the IV ventricle represent 1–5% of all intracranial lesions (1–4); among tumors of the ventricular system, they are diagnosed in 2/3 of cases (3). According to the modern standards, the first stage of treatment for this pathology is microsurgical removal (5). However, surgical treatment of these tumors remains a challenge for neurosurgeons due to the proximity of functionally important anatomical structures (the brainstem, the cerebellum, pathways, vessels, etc.) of the posterior cranial fossa. Therefore, surgery in this area is associated with the possible occurrence of a wide range of postoperative complications. To date, despite the general trend toward minimally invasive surgery, the standard median suboccipital approach is commonly used for removing tumors of the IV ventricle and brainstem. According to the literature, a median suboccipital approach to the posterior cranial fossa is associated with a high risk of CSF leakage (up to 27% of cases) and pseudomeningocele (up to 23% of cases) (6). The goal of this paper is to review the different variations of the median suboccipital approaches used to surgically treat fourth ventricular pathologies. A summary of the analyzed articles can be seen in Table 1.

**Table 1:** Analyzed series of the surgically treated patients with 4 ventricular and brainstem tumors.

Year	1997	1999	2001	2002		2003	2004	2004	2007	2010	2012	2013	2015	2016	2018	2018	2018	2018	2018
Authors	Kellogg (46)	Ziyal (27)	Matsushima (9)	Gnanalingham (6)		Jean (28)	Gok (25)	El-Bahy (30)	Rajesh (29)	Zaheer N.S. (24)	Karakhan (3)	Han (15)	Tomasello (21)	Bo Qiu (31)	Ferguson (2)	Kalinovsky (23)	Babichev (22)	Kushel (26)	Pitskhelauri (47)
				Craniectomy	Craniotomy														
Average patients age	N\A	49.2 (23–63)	25.1 (1–70)	0.3–15 (5.56)	0.2–14 (5.96)	52 (26–69)	20 (1–56)	20.6 (5–45)	Under 10 y.o.	8 (1–17)	16–68	23 (6–58)	22.5 (2–66)	8.7 (2.5–14)	35.5	39.3 (19–61)	37.7 (32.8–42.4)	N\A	36 (17–66)
Patient position	Prone	Semi-prone	N\A	Sitting 98%	Lateral decubitus	Sitting	N\A	Prone, oblique	Prone	N\A	Prone	Prone	Prone	70% Lateral	Semisitting	Sitting	Sitting	Sitting 67.1%	
Osteoplastic craniotomy	N\A	N\A	N\A	0%	100%	N\A	N\A	N\A	N\A	100%	N\A	N\A	0%	5 × 4 cm	44 (80%)	100%	N\A	100%	95.7%
Craniectomy	N\A	N\A	N\A	100%	0%	N\A	N\A	N\A	100%	0%	N\A	N\A	100%	5 × 4 cm	11(20%)	0%	N\A	N\A	4.3%
Resection of C1 arch	N\A	N\A	N\A	4%	4%	100%	N\A	N\A	N\A	N\A	N\A	9 (18%)	15 (33%)	N\A	N\A	13 (39.4%)	56.10%	N\A	11 (15.7%)
Dural incision	Y-shaped	Y-shaped	N\A	N\A	N\A	Y-shaped	Y-shaped	N\A	N\A	U-shaped	N\A	Y-shaped	Y-shaped	Y-shaped	N\A	U-shaped	Y- or U-shaped	V- or Y- shaped	Y-shaped
Telovelar approach	Cerebello-medullary	Subtonsillar-transcerebellomedullary	N\A	N\A	N\A	N\A	Trans-cerebellomedullary	100%	100%	100%	92.9% (in other cases - supracerebellar)	36%	100%	19.20%	26 (48%)	100%	16 (39%)	N\A	N\A
Unilateral approach	N\A	N\A	31.60%	N\A	N\A	N\A	100%	N\A	N\A	N\A	N\A	N\A	N\A	17 (65.4%)	N\A	N\A	N\A	N\A	N\A
Bilateral approach	N\A	N\A	68.40%	N\A	N\A	N\A	N\A	N\A	N\A	N\A	N\A	N\A	100%	9 (34.6%)	N\A	N\A	N\A	N\A	N\A
Median aperture approach	N\A	N\A	N\A	N\A	N\A	N\A	N\A	N\A	N\A	N\A	N\A	64%	N\A	80.80%	N\A	0%	25 (61%)	N\A	100%
Transvermian approach	N\A	N\A	N\A	N\A	N\A	N\A	N\A	0%	N\A	0%	N\A	N\A	N\A	N\A	24 (44%)	0%	N\A	N\A	N\A
Shunting procedures preop	N\A	N\A	N\A	N\A	N\A	1 (33.3%)	N\A	N\A	N\A	6 (30%)	0	7 (14%)	N\A	3 (11.5%)	16%	N\A	12 (29.3%)	N\A	N\A
Shunting procedures intraop	N\A	N\A	N\A	N\A	N\A	N\A	N\A	N\A	N\A	N\A	N\A	N\A	N\A	9 (34.6%)	14 (25%)	N\A	N\A	154 (73%)	N\A
Shunting procedures postop abs (%)	N\A	50%	N\A	23%	15%	N\A	N\A	N\A	N\A	6 (30%)	N\A	7 (14%)	2(4.5%)	1 (3.8%)	12 (22%)	1 (3%)	N\A	2 (0.95%)	0%
Hematomas	N\A	N\A	N\A	N\A	N\A	N\A	1 (4.8%)	N\A	N\A	N\A	N\A	N\A	N\A	N\A	1 (2%)	1 (3%)	N\A	5 (2.4%)	N\A
Meningitis	N\A	N\A	N\A	20%	11%	N\A	N\A	N\A	N\A	N\A	N\A	N\A	N\A	N\A	3 (5%)	N\A	1 (2.4%)	1 (0.47%)	N\A
CSF leak abs (%)	N\A	N\A	N\A	27%	4%	N\A	N\A	N\A	N\A	2 (10%)	N\A	N\A	N\A	N\A	4 (7%)	0%	4 (9.8%)	6 (2.8%)	11.4%

### Main Surgical and Anatomical Landmarks of the Suboccipital Cerebellar Surface

When removing tumors of the IV ventricle, it is essential to know the anatomical structures and landmarks of the suboccipital surface of the cerebellum, especially its fissures. The tonsillobiventral fissure is located between the upper-lateral border of the tonsil and the biventral lobule. During surgery, the dissection of this fissure is performed via the supratonsillar approach (7, 8).

The lower-medial surface of the tonsil is divided from the medulla and the uvula by the cerebellomedullar fissure, which consists of the tonsillomedullar and tonsillouvular fissures. The anterior wall of the cerebellomedular fissure is formed by the posterior surface of the medulla, the inferior medullary velum, and the tela choroidea. The posterior wall is formed by the uvula, the tonsil, and the biventral lobule laterally (9–11).

The floor of the tonsillouvular fissure is adjacent to the inferior half of the IV ventricular roof. The inferior half of the roof is formed by the inferior medullary velum, the tela choroidea, the nodule and the uvula. The inferior medullary velum is a butterfly shaped structure that blends into the ventricular surface of the nodule medially (12). It is a membranous layer and does not contain eloquent nervous tissue (13, 14). At the fastigium, it bends inferiorly from the superior medullary velum and blends with the tela choroidea at the level of the lateral recesses. The tela choroidea is a triangular fold of the pia mater (13, 15–17).

### Different Approaches to Classifying Fourth Ventricular Tumors

To date, there is no commonly accepted classification system for fourth ventricular tumors. The main controversy is whether to classify tumors that spread secondary to the ventricular cavity and deform the it as tumors of the IV ventricle. In almost half of the cases, the origin site of tumor growth is the floor of the IV ventricle (3).

In the publications from the beginning of the 20th century, the prevailing opinion was that, from an anatomical point of view, IV ventricle tumors should include only those located in its cavity, i.e., growing from its floor or ependyma of the vascular plexus, according to articles of M. Yu Rappoport, I. Ya Razdolsky, Hennenberg, Cushing, O. Marburg, and Lerrebuhle (18, 19).

The majority of contemporary authors expand the concept of the term “tumor of the IV ventricle”. For instance, Yasargil in his seminal work in 1994 classified both those tumors that develop primarily in its cavity (subependymoma, ependymoma, choroidpapilloma) and those tumors that grow secondarily to its cavity (astrocytomas, medulloblastomas, etc.) as IV ventricular tumors (20). Karakhan (3) and Tomasello (21) built their classifications of IV ventricular tumors according to the same principle (intraventricular filling and subependymal protrusion).

Yasargil distinguishes tumors of the IV ventricle according to the topographic localization of the tumors, which occupy the upper-median/paramedian, lower-median/paramedian, posterior, and lateral locations (20).

In their publications, Ferguson and Babichev divided IV ventricular tumors into those with central, caudal, lateral, and oral brainstem distributions (2, 22). Karakhan divided IV ventricular neoplasms into intraventricular, with supra-, retro-, infra- and latero-compression (3) (Table 2).

**Table 2:** IV ventricle tumor classifications.

Year	Author	IV ventricle tumor classification
1935	M.Y. Rappoport (18)	Lesion located in the IV ventricular cavity, growing from its floor, ependyma of the choroid plexus
1940	I.Y. Razdolsky (19)	Lesion developed from the walls of the IV ventricle (ependyma and subependymal glia), endothelium, stroma, blood vessels, choroid plexus, ventral cerebellar vermis.
1994	M.G. Yasargil (20)	I. I Lesions grow primarily within the IV ventricle II. II Lesions secondarily spread into the IV ventricular cavity
		Topographically: 1) Upper median and paramedian location 2) Lower median and paramedian location 3) Posterior (fastigial) location 4) Lateral location
2018	K.N.Babichev (22)	In relation to the floor of the IV ventricle: 1) Central extension 2) Caudal extension (through the median aperture) 3) Lateral extension (through the lateral aperture into the cerebellar-pontine cistern) 4) Oral-brainstem extension (infiltrating the brainstem) 5) Mixed
2015	V.B. Karakhan (3)	In relation to the IV ventricular cavity: 1) Intraventricular filling 2) Extraventricular compression (subependymal protrusion)
		Topographic variants: 1) Intraventricular 2) Supraventricular compression (from the superior medullary vellum) 3) Retroventricular compression (from the inferior medullary vellum) 4) Infraventricular compression (from the brainstem) 5) Lateroventricular compression (from the lateral aspects of the medulla)
2018	S.D. Ferguson (2)	1) No extension beyond the 4th ventricle 2) Caudal extension (into the foramen of Magendie) 3) Lateral extension (into the foramen of Luschka) 4) Anterior extension (invasion of the brainstem) 5) Extension in multiple directions

The most common tumor types in the IV ventricle and brainstem are medulloblastomas (2, 10, 15, 21–26) (7–93.3%), metastases (2, 3, 21, 25, 27, 28) (4.8–46.4%), ependymomas (2, 3, 9, 15, 21–30) (6.7–38%), astrocytomas (grade I–IV) (2, 3, 9, 21–26, 28, 30) (7.3–33%), subependymomas (2, 22) (9–19.5%), chorioidpapillomas (2, 9, 21–23, 26, 30) (2.2–14.6%), and hemangioblastomas (2, 3, 9, 15, 21–23, 25) (4.9–14.3%).

### The Clinical Picture of Tumors of the IV Ventricle

The symptoms that occur in patients with tumors of the fourth ventricle are either a consequence of an increase in intracranial pressure due the restriction in the outflow of cerebrospinal fluid through the cavity of the 4th ventricle or the compression of the walls of the 4th ventricle by the tumor (4).

For this reason, tumors of the IV ventricle are characterized by symptoms of increased ICP (headache, nausea, vomiting); symptoms of cerebellar damage, in particular, ataxia, intentional tremor, cerebellar dysarthria; and dysfunction of the cranial nerves (from V to XII). Motor and sensory disorders are less common and occur in cases where there is significant size and spread of the tumor (19).

According to the analyzed series, the most common symptoms were headache (2, 22, 28, 30, 31) (33–100%), nausea, vomiting (2, 22, 28, 31) (38–100%), diplopia (27, 28, 31) (26.9–33.3%), gait disturbance (2, 22, 27, 28, 30) (33–100%), visual impairment (2, 22) (9.8–29%), dizziness (2, 22, 28, 31) (15.4–66.7%), bulbar syndrome (22, 27, 28, 31) (17, 1–50%), sensory and pyramidal disorders (2, 22, 23, 31) (7–69.2%) and changes in mental status (2, 22, 31) (2–17.2%).

### Surgical Treatment of Tumors of the IV Ventricle

In the 1920s, the only methods of surgical intervention for tumors of the fourth ventricle were punctures of the lateral ventricles, punctures of the corpus callosum, and subtemporal decompressive trepanations. These manipulations were palliative and did not produce favorable results.

The first surgical operation for a tumor of the IV ventricle was performed by Germanides (1894), and the first successful surgical interventions were performed by Oppenheim (1912) and Krause (1913). Cushing developed a surgical technique for these pathologies, which consisted of a wide exposure of both halves of the posterior cranial fossa. Martel began to operate on such patients while they were sitting and with drainage of the surgical wound (19). However, the overall mortality in pathologies of the fourth ventricle at the beginning of the 20th century remained high. For example, in the work of Razdolsky I.Ya, the postoperative mortality was 31% (19).

To date, along with the development of microsurgical techniques, anesthesia, new methods of intraoperative neurophysiological monitoring, and a detailed study of the anatomy of the 4th ventricle, the results of surgical treatment have improved significantly, but nevertheless, treating this pathology is a difficult task for modern neurosurgeons.

### Variants of the Neurosurgical Approaches to Tumors of the 4th Ventricle

#### Craniotomy Stage

The classic approach to tumors of the 4th ventricle is the suboccipital approach (32). After the abovementioned approach is performed, the cerebellar hemispheres, vermis and medulla are exposed. Compared to keyhole approaches, this technique has advantages and disadvantages. The advantages are that there is exposure of more anatomical structures and, thus, better orientation in the surgical wound and freedom of manipulation. However, this approach is associated with large muscle and soft tissue incisions, large exposure of the brain tissue to environmental influences (microscope light, etc.) and an increased risk of sinus damage. The authors prefer both resection (21, 29) and osteoplastic (23, 24, 26) craniotomy. Both options have advantages and disadvantages; however, according to Gnanalingham’s study that compared the complications of craniotomy and craniectomy, the number of CSF leaks and pseudomeningoceles was significantly higher in the craniectomy group of patients (27 vs. 4% and 23 vs. 9%, respectively) (6).

The literature also describes a suboccipital median approach with an attached flap (33). The essence of the method lies in the fact that when performing a craniotomy, a bone flap is left on the atlantooccipital membrane. When the wound is closed, the bone is fixed into place more easily and is more stable. According to the authors, this technique reduces the risk of CSF leakage and postoperative headaches due to better preservation of the anatomy of the atlantooccipital joint and the natural soft tissue layers.

If the tumors are located below the Chamberlain line, it is possible to perform a resection of the C1 arch (13, 15, 22, 23).

When analyzing the latest published works, one can note a clear trend toward an increase in the use and indications for minimally invasive approaches (34–36).

Several clinical cases of median keyhole approaches to the IV ventricle have been described. Among them, depending on the “entrance gate” to the IV ventricle, two types of surgeries are distinguishable: a) the median suboccipital approach (34–37) and b) the approach through the posterior atlanto-occipital membrane without bone resection (38).

Some authors believe that in the absence of tumor spread to the cranial parts of the IV ventricle at the micro stage, it is possible to apply an approach through the median aperture by cutting the tela choroidea from the foramen of Magendie laterally without cutting the inferior medullary velum upward.

Bo Qiu’s article described the removal of the ependymomas of the IV ventricle in several cases, and in 80.8% of the cases, it was enough to cut the tela choroidea for adequate removal of the tumors. He also believes that with an approach through the median aperture, with proper retraction of the tonsils and uvula, one can clearly visualize the lateral recess and the lower ¾ of the cavity of the IV ventricle (31). Due to the uselessness of performing an upward incision of the inferior medullary velum and the choice of the optimal trajectory, the size of the craniotomy can be significantly minimized.

Suboccipital keyhole approaches allow the exposure of the tonsils, vermis and medulla. They provide a smaller soft tissue incision, less risk of traction of the cerebellum and other anatomical structures, less environmental impact (microscope light) and less risk of sinus damage. At the same time, when performing a keyhole approach, the surgeon is limited in manipulations and is required to have high qualification and experience.

In his monograph “Keyhole approaches in neurosurgery” in 2008 (34), Perneczky described a midline suboccipital keyhole technique with a working corridor diameter of 3 cm, including the space above the posterior atlantooccipital membrane. Perneczky also proposed variations of the suboccipital approach depending on the localization of the tumor in the cavity of the IV ventricle (34).

Charlie Teo described a keyhole approach to the IV ventricle using a mini-telovelar approach, suggesting that the cerebellar hemispheres do not always need to be fully exposed and that reducing the size of the craniotomy reduces the amount of laterally dissected muscles. In his opinion, the bone should only be resected until the border of trepanation is level with the upper pole of the tumor of the IV ventricle (35).

In 2019, David Pitskhelauri published a series of 200 patients who had surgery using the burr-hole technique, and a minimally invasive suboccipital approach was used in 17 patients. The size of the craniotomy was 14 mm, and the length of the incision was 3–4 cm (36).

In 2020, Corniola described a clinical case of microsurgical removal of a small subependioma of the caudal parts of the IV ventricle using a 1 cm resection of the occipital bone (37).

The median minimally invasive suboccipital approach, in addition to being used for the removal of tumors of the posterior cranial fossa with a median localization, is also used for the treatment of other pathologies of the central nervous system.

Hamada (39) used a small craniotomy, and Rauf (40) performed an endoscopic aqueductoplasty in an isolated IV ventricle through a suboccipital burr hole, 2–3 cm below the superior nuchal line. Toyota (41), with a small craniotomy, and Gallo, using a 4 cm incision and minimal resection of the occipital bone (42), used an endoscopic approach into the isolated IV ventricle to perform aqueductoplasty.

It is worth noting the work of Bergsneider (38), which described an endoscopic approach to the IV ventricle through the foramen magnum without bone resection. The author successfully used a flexible endoscope to remove cysticercus cysts in the IV ventricles of 5 patients.

#### Micro Stage

The first approach to the IV ventricle is considered to be the transvermian approach, which was described by Walter Dandy. It consisted of dissection of the cerebellar vermis (43). However, its application is currently limited due to the significant neurological deficits that are caused by this approach (44).

Currently, different variations of the approach to tumors of the IV ventricle through the cerebellar-medullar fissure, as described by Matsushima et al., have been generally accepted (16). In terms of surgical significance, he compared the cerebellomedullar fissure in the subtentorial space with the Sylvian fissure in the supratentorial space. Depending on the location of the tumor in the IV ventricle, Matsushima et al. subdivided the approach through the cerebellomedullary fissure into several methods. The “extensive opening method” is used when tumors are localized deep in the cavity of the IV ventricle or are paramedian near the cerebral aqueduct. In this case, both the uvulotonsillar and medullotonsillar spaces are dissected bilaterally. The “lateral wall opening method” involves a unilateral dissection of the cerebellomedullar fissure to access the central areas of the IV ventricle. In the case of lateralization of a mass lesion, the authors recommended dissecting the contralateral fissure. With the “lateral recess opening method”, in cases where the tumor is located in the most distal parts of the lateral recess, a unilateral dissection of the tonsillomedullar fissure is used (9).

Additionally, one of the approaches to the IV ventricle is through the foramen of Magendie, which is often enlarged by the tumor, and with situational dissection of the tela choroidea (22).

There is also a transcortical approach to the IV ventricle through the cerebellar hemisphere using a tubular retractor, which was described by Jamshidi et al. in a series of 3 patients. Taking into account the data of the navigation system, the surgical corridor is built in such a way as not to damage the deep nuclei of the cerebellum (45).

Most authors currently use variations of the cerebellomedullar fissure approach (3, 9, 21, 23, 24, 27, 29, 30, 46). In some series, the authors have used both of the approaches through the cerebellomedullar fissure and through the foramen of Magendie with dissection of the choroid plexus (15, 22, 31). Ferguson et al. published a series of patients who had surgery that utilized the telovelar and transvermian approaches (2). It can be noted that there is a growing trend toward the use of approaches through the cerebellomedullar fissure and a departure from the transvermian approach, which is used situationally for tumors originating from the cerebellar vermis.

### Complications of Surgical Treatment

In most of the described case series, the symptoms of neurological status deterioration in the postoperative period have included the following: general worsening of neurological symptoms (2, 21–23, 29) (4.5–46.7%), cerebellar mutism (2, 15, 21, 23, 24, 29–31) (0–38%), worsening of bulbar disorders (2, 21–23, 25, 28) (2.2–38%), oculomotor disorders (21, 23, 25, 27, 29, 46) (4.5–20%), gait disturbances (2, 22, 28–30) (25–56%), and facial nerve deficits (23, 25, 27, 29, 46) (3–18.2%).

Postoperative hematomas have been described in 2–4.8% of cases (2, 23, 25, 26).

The incidence of CSF leakage in the analyzed series varied from 0 to 27% (2, 6, 22–24, 26, 47), and the incidence of postoperative meningitis was noted in 0.47% - 20% of the cases (2, 6, 22, 26).

During the perioperative period, intraoperative air embolism occurred as a complications in 9.5% of patients who had surgery while sitting in the study by Gok et al. (25). Symptomatic pneumocephalus was observed in two series of patients with an incidence of 4 and 0.95%, where the position of the patients was predominantly on their side and when sitting, respectively (2, 26).

In the analyzed series, postoperative CSF shunting operations due to unresolved occlusive hydrocephalus were needed in 0–50% of the cases (2, 6, 15, 21, 23, 24, 26, 27, 31, 47).

## Conclusion

Despite the complexity of the anatomy of the fourth ventricle of the brain, recent studies have shown that microsurgical removal of tumors of this localization is feasible with an acceptable incidence of postoperative neurological deficits and surgical complications.
